# *Torreya grandis* Diester Oil Attenuates High-Fat Diet-Induced Pulmonary Inflammation with Superior Efficacy to Natural *Torreya grandis* Oil

**DOI:** 10.3390/nu18111671

**Published:** 2026-05-23

**Authors:** Lixia Jia, Hongling Lu, Chenkai Jiang, Wenjun Hu, Ganglei Yu, Xingwei Xiang, Guoxin Shen, Jing Tao, Lin Chen, Wenhua Miao

**Affiliations:** 1 College of Food and Pharmacy, Zhejiang Ocean University, Zhoushan 316022, China; 19818004818@163.com (L.J.); miaowenhua@126.com (W.M.); 2 Institute of Sericultural and Tea, Zhejiang Academy of Agricultural Sciences, Hangzhou 310021, China; luhongling@zaas.ac.cn (H.L.); greenbreezekai@126.com (C.J.); guyuexingshi@163.com (W.H.); guoxin.shen@ttu.edu (G.S.); 3 Dongyang Xiangfei Technology Co., Ltd., Jinhua 322100, China; 13505890898@163.com; 4 College of Food Science and Technology, Zhejiang University of Technology, Hangzhou 310014, China; 5 Zhejiang Forest Resources Monitoring Center, Hangzhou 310020, China

**Keywords:** obesity, *Torreya grandis*, diester oil, lung, inflammation, microbiome

## Abstract

Background/Objectives: A high-fat diet (HFD) not only induces metabolic disorders but also causes oxidative damage to the lung tissue, triggering inflammatory responses. However, the detailed mechanisms by which HFD induces pulmonary oxidative stress and inflammation, particularly involving NF-κB/PPAR-γ signaling and lung microbiota, remain poorly understood, and effective dietary intervention strategies are still lacking. This study investigated the effects of HFD on lung tissue injury in mice and systematically evaluated the protective effects and potential mechanisms of *Torreya grandis* seed oil (TGO) and *Torreya grandis* seed diester oil (TGO-DG). Methods: After 12 weeks of HFD feeding, HFD group mice exhibited a marked increase in body weight (90.36%) compared with the control group, whereas body weight gain was significantly attenuated in the TGO (57.95%) and TGO-DG (55.78%) groups. Results: Biochemical analyses revealed that the levels of malondialdehyde (MDA), nitric oxide (NO), and pro-inflammatory cytokines (TNF-α, IL-6, IL-1β) were significantly elevated in the HFD group, indicating pronounced oxidative stress and inflammatory responses in lung tissue. These symptoms were significantly attenuated by TGO and TGO-DG, with TGO-DG showing a more marked effect. Western blot (WB) results showed that both TGO and TGO-DG suppressed IL-6 expression and altered the expression of proteins in the NF-κB and PPAR-γ signaling pathways, which may contribute to the alleviation of pulmonary inflammation. Lung microbiota analysis revealed that TGO was associated with an increased proportion of *Lactobacillus* species, which correlated with the restoration of pulmonary microbial homeostasis. Conclusions: Overall, these results suggest that TGO and TGO-DG effectively alleviate HFD-induced oxidative stress and inflammation in lung tissue through regulation of inflammatory signaling pathways and lung microbiota composition. Notably, TGO-DG exhibited superior protective effects, highlighting its potential as a lipid ingredient.

## 1. Introduction

Obesity has become a major global public health challenge, and its prevalence has increased steadily in recent years, primarily driven by unhealthy dietary patterns such as long-term consumption of high-fat diets (HFDs) [1]. HFD-induced obesity has been considered a critical risk factor for metabolic syndrome, non-alcoholic fatty liver disease, and cardiovascular diseases [2]. In recent years, accumulating evidence has further revealed that obesity also substantially contributes to the development of respiratory diseases. Obesity can induce a distinct pathological condition termed “obesity-associated lung injury”, which is characterized by chronic low-grade inflammation and oxidative stress in lung tissue [3]. Despite growing recognition of this condition, effective and targeted intervention strategies for obesity-related lung injury remain limited. Therefore, identifying safe and effective protective agents derived from natural products and functional foods is of considerable scientific and practical significance.

*Torreya grandis* seed, a rare dried nut endemic to China, is rich in oils predominantly composed of unsaturated fatty acids [4]. *The Chinese Pharmacopoeia* records its traditional uses for eliminating intestinal parasites, relieving digestive discomfort, and promoting expectoration and bowel movements [5]. In addition, extracts from various parts of *T. grandis* have been shown to have pharmacological activities such as anti-inflammatory, antibacterial, and hypoglycemic effects. This species holds significant nutritional and medicinal value. *T. grandis* seed is rich in oils (42.6–61.5%), proteins (10.34–16.43%), carbohydrates (10.79–21.42%), and vitamins and minerals. Due to its multiple bioactivities, including antioxidant, antibacterial, hypolipidemic, anti-inflammatory, and hypoglycemic properties, *T. grandis* represents a valuable nutritional and health supplement to the human diet. However, the potential protective effects of *Torreya grandis* oil (TGO) and its diester derivative (TGO-DG) on obesity-induced lung injury have not yet been systematically investigated.

Excessive fat intake is a major contributor to the development of various chronic metabolic diseases [6]. Among dietary lipids, glycerol diester oils have garnered significant attention due to their distinct metabolic pathway compared with conventional triglyceride oils. During metabolism, glycerol diester oils are less prone to storage in adipose tissue and are preferentially oxidized to generate energy, thereby exerting multiple beneficial physiological functions, including regulation of lipid metabolism, inhibition of body weight gain, and improvement of glucose homeostasis [7]. Previous studies have demonstrated that edible oils rich in glycerol diesters can effectively prevent hyperlipidemia induced by obesity in both animal models and humans [8]. As a structural lipid derived from TGO, *Torreya grandis diester oil* (TGO-DG) may exhibit enhanced metabolic regulatory potential compared with conventional oils [9,10]. However, studies have mainly focused on the regulatory effects of these components on hepatic or intestinal metabolism, while their protective role in distant organs such as the lung, particularly under HFD-induced obesity, remains largely unexplored.

Long considered sterile, human lungs have now been confirmed to harbor a complex and dynamic microbial community known as the lung microbiome [11]. Imbalances within this community are closely associated with the onset and progression of various chronic and acute pulmonary diseases. The lung microbiome undergoes changes in numerous pulmonary disorders, including chronic conditions such as chronic obstructive pulmonary disease (COPD), asthma, and bronchiectasis; acute illnesses such as pneumonia, sepsis, and COVID-19; and other pulmonary complications associated with lung transplantation, lung cancer, and HIV/AIDS. The role of the lung microbiome in regulating host immunity and inflammation within the lungs and distant organs is increasingly understood [12]. Research indicates that the core mechanism lies in the interaction between microbes and the host immune system. For example, specific microbial communities can influence the tumor microenvironment through their metabolic products or directly drive pulmonary fibrosis via specific receptor signaling pathways.

To date, increasing attention has focused on the role of the microbiome in host metabolic and immune regulation [13,14,15]. However, findings regarding obesity-associated microbial alterations remain highly heterogeneous. Furthermore, little information is available regarding the effects of obesity on the microbial composition of the lung or the potential involvement of the gut-liver-lung axis. Therefore, a comprehensive investigation into lung microbiota alterations during HFD-induced obesity is warranted.

Based on these considerations, the present study aimed to establish a mouse model of HFD-induced pulmonary inflammation and to systematically evaluate the protective effects of TGO and TGO-DG against lung inflammation. Potential mechanisms were explored from multiple dimensions, including oxidative stress, inflammatory responses, the lung microbiome, and the regulation of key signaling pathways such as NF-κB and PPAR-γ. This study provides new insights into the development of functional foods targeting obesity-related lung inflammation.

## 2. Materials and Methods

### 2.1. Materials and Reagents

*Torreya grandis oil* and *Torreya grandis* diester oil were prepared and provided by the Sericulture and Tea Research Institute of Zhejiang Academy of Agricultural Sciences (Hangzhou, China). Superoxide dismutase (SOD), malondialdehyde (MDA), and nitric oxide (NO) assay kits were supplied by Nanjing Jiancheng Technology Co., Ltd. (Nanjing, China). Tumor Necrosis Factor-alpha (TNF-α), interleukin-6 (IL-6), and interleukin-1β (IL-1β) assay kits were purchased from Wuhan Boshide Biotechnology Co., Ltd. (Wuhan, China). RIPA lysis buffer was obtained from Biyuntian Biotechnology Co., Ltd. (Shanghai, China). The RNA Easy Fast Tissue/Cell Kit, FastKing gDNA Dispersing RT SuperMix Kit, and FastReal qPCR PreMix (SYBR Green) Kit were supplied by TIANGEN (Beijing, China). BCA protein quantification kit and ECL chemiluminescence substrate were purchased from Haoke Biotechnology Co., Ltd. (Hangzhou, China).

### 2.2. Experimental Animals and Grouping

Forty SPF-grade C57BL/6J mice (body weight 18–22 g) were purchased from Hangzhou Ziyuan Laboratory Animal Technology Co., Ltd. (Hangzhou, China). All mice were housed in a standard animal facility (temperature 23 ± 2 °C, relative humidity 60 ± 5%, 12-h light/dark cycle) with free access to food and water. This animal study protocol was approved by the Zhejiang Academy of Agricultural Sciences Laboratory Animal Ethics Committee (Approval No.: 2022ZAASLA86). After one week of acclimation feeding, mice were randomly divided into 5 groups of 8 mice each: the control group (C), model group (M), positive control group (S), *Torreya grandis* seed oil (TGO) group, and *Torreya grandis* diester oil (TGO-DG) group. Group C received standard chow, while groups M, S, TGO, and TGO-DG were fed a high-fat diet (a high-calorie diet constituting approximately 45% of total caloric intake). Additionally, at the same time each day, mice in the M group received 2.5 mL/kg soybean oil via gavage; mice in the S group received 2.5 mL/kg soybean oil containing 2 mg/kg lovastatin via gavage; mice in the TGO group received 2.5 mL/kg *Torreya grandis* oil via gavage; and mice in the TGO-DG group received 2.5 mL/kg *Torreya grandis* diester oil via gavage. The C group received an appropriate volume of physiological saline based on body weight.

Mice were randomly assigned to five groups (*n* = 8 per group) using a computer-generated random number sequence (Excel, Microsoft Corp., Redmond, WA, USA). The randomization list was generated by a researcher not involved in animal handling or data collection.

Blinding: Animal housing, gavage administration, and sample collection were performed by one investigator, while outcome assessments (biochemical assays, RT-PCR, Western blot, and microbiota analysis) were conducted by another investigator blinded to the group allocation. Data analysis was also performed blinded.

Sample size calculation: The choice of *n* = 8 per group was based on previous studies in the field [16], which have consistently demonstrated that this sample size is sufficient to detect significant differences in HFD-induced pulmonary inflammation endpoints. While a formal a priori power calculation was not performed, the observed statistical differences in our study (e.g., *p* < 0.05 for multiple comparisons) further support the adequacy of the sample size.

### 2.3. Sample Collection

During the rearing period, weekly records of mouse food intake and body weight were maintained. The entire feeding process lasted 12 weeks, after which mice were fasted for 12 h. They were deeply anesthetized with 1% pentobarbital (30 mg/kg, i.p.). Once unresponsive to toe pinch, euthanasia was performed by exsanguination via the inferior vena cava. The lungs were rapidly dissected, harvested, immediately flash-frozen in liquid nitrogen, and then transferred to an −80 °C ultra-low-temperature freezer for subsequent biochemical and molecular analyses.

### 2.4. Measurement of Mouse Body Weight Gain Rate, Lung Weight, and Lung Index

During the rearing period, food intake and body weight of the mice were recorded weekly. On the final day, the lungs were removed, rinsed with sterile PBS, blotted with filter paper to remove residual solution, weighed sequentially, and the weights were recorded. The lung index was calculated based on the lung weights using the formula:

$$\mathrm{Lung}\;\mathrm{Index}\;=\;\frac{\mathrm{Lung}\;\mathrm{weight}}{\mathrm{Mouse}\;\mathrm{body}\;\mathrm{weight}}\times 100$$

### 2.5. Biochemical Indicator Testing

A 0.2 g sample of the right lung lobe was weighed and homogenized in a tissue homogenizer. Nine volumes of physiological saline were added to prepare a 10% (*m*/*v*) lung tissue homogenate. The homogenate was centrifuged at 12,000× *g* rpm for 10 min at 4 °C, and the supernatant was collected. The levels of inflammatory cytokines, including tumor necrosis factor-α (TNF-α), interleukin-6 (IL-6), and interleukin-1β (IL-1β), in the lung homogenate were detected using an enzyme-linked immunosorbent assay (ELISA) kit following the manufacturer’s instructions [17,18].

The Nanjing Jiancheng assay kit was used to detect oxidative stress markers in lung tissue homogenates: superoxide dismutase (SOD) activity, malondialdehyde (MDA) content, and nitric oxide (NO) concentration.

### 2.6. Real-Time Quantitative PCR Analysis

Total RNA was extracted from frozen lung tissue using the Tiangen kit. RNA was reverse transcribed into cDNA using a reverse transcription kit. mRNA expression levels of genes related to the NF-κB, PPAR-γ, and IL-6 signaling pathways in lung tissue were detected using the SYBR Green method on a real-time quantitative PCR instrument. β-actin was used as the internal reference gene, and the relative expression levels of genes were calculated using the 2^−ΔΔCt^ method. The primer sequences are provided in Supplementary Materials.

### 2.7. Protein Western Blot Analysis

Mouse lung tissue was thoroughly homogenized and transferred to a 1.5 mL centrifuge tube. The sample was sonicated on ice (200 W, 15% power, 3-s pulses with 5-s intervals for 3 min). The supernatant was collected by centrifugation at 12,000× *g* rpm for 15 min at 4 °C. Protein concentration was measured using a BCA protein quantification kit. Proteins were denatured and subjected to SDS-PAGE gel electrophoresis, with equal volumes (20 µg) loaded per well. After electrophoresis, proteins were wet-transferred to PVDF membranes. Following membrane blocking, primary antibodies against NF-κB p65, PPAR-γ, IL-6, and β-actin were incubated overnight at 4 °C. Subsequently, the membranes were incubated with the corresponding secondary antibodies. After washing, the protein bands were visualized using ECL chemiluminescence. Images were observed and recorded using an imaging system. The images were processed using Photoshop software (version 23.4.1). Grayscale values of the target bands were analyzed using ImageJ software (version 1.8.0). β-actin was used as the internal control. The relative expression levels of target proteins were calculated.

### 2.8. 16S Analysis of Pulmonary Microbiota

According to L. J. Zheng, C. J. Liu, et al., extract microbial genomic DNA from lung tissue [19]. After completing genomic DNA extraction, detect the extracted genomic DNA using 1% agarose gel electrophoresis. Perform PCR amplification of the V3-V4 hypervariable region of the bacterial 16S rRNA gene using diluted genomic DNA as a template, and sequence it on the Megi platform. Sequencing data undergo operational taxonomic unit (OTU) clustering followed by bioinformatics analysis of community diversity and structure. To minimize and monitor potential contamination due to the low microbial biomass of lung tissue, the following measures were implemented: (i) sterile phosphate-buffered saline (PBS) was processed alongside lung samples as a negative control through all steps (DNA extraction, PCR amplification, and sequencing); (ii) no-template controls (NTCs) were included in each PCR run; (iii) all instruments and bench surfaces were cleaned with 70% ethanol and UV-treated before use; (iv) DNA extraction and PCR setup were performed in a laminar flow hood dedicated to low-biomass samples. Any operational taxonomic units (OTUs) detected in negative controls were subtracted from the experimental samples. Sequencing quality was assessed using the following criteria: reads with an average quality score < Q20 were trimmed; chimeric sequences were removed using USEARCH (v11); OTUs with abundance < 0.001% of total reads were filtered out. Only samples with >10,000 high-quality reads were retained for downstream analysis.

### 2.9. Statistical Analysis

All data are expressed as mean ± standard deviation. Data analysis was performed using SPSS Statistics 27 software. One-way analysis of variance (ANOVA) was conducted. Duncan’s multiple range test was used for post hoc comparisons between groups. GraphPad Prism 10.1.2 software was used for data visualization. Differences were considered statistically significant at *p* < 0.05.

## 3. Result and Discussion

### 3.1. Mouse Body Weight Gain Rate, Lung Weight, and Lung Index

**Figure 1:**
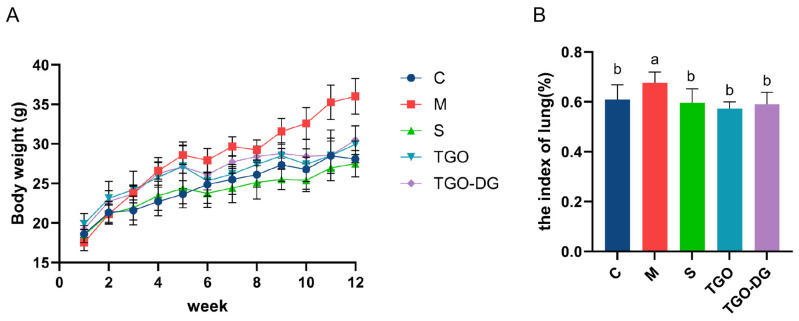
Weight gain rate, lung weight, and lung index in high-fat diet mice (**A**) Weight changes, (**B**) Lung index. Different letters within the same column indicate significant differences (*p* < 0.05).

TGO has been reported to exhibit anti-obesity, neuroprotective, anti-diabetic, and constipation-relieving effects [20,21,22]. Weight monitoring results are shown in Figure 1A. After the first week of adaptive feeding, all groups exhibited a gradual increase in body weight, indicating normal feeding status. Using body weight at the end of the first week as the baseline, mice in the model group exhibited a marked and continuous weight increase throughout the experimental period. At the end of 12 weeks, mice in the control (C) and positive control (S) groups showed a stable increase, while mice in the model group exhibited a significant body weight gain of 90.36%, confirming successful HFD-induced obesity. Mice in the TGO and TGO-DG intervention groups also exhibited reduced body weight gain compared with the model group, with increases of 57.95% and 55.78%, respectively, indicating a moderate alleviating effect on HFD-induced weight gain. Furthermore, the lung index of mice in the model group was significantly higher than that of the other groups (Figure 1B), suggesting notable differences in lung mass relative to body weight. Treatment with lovastatin, TGO, or TGO-DG significantly reduced the lung index compared with the model group, suggesting that these interventions mitigated HFD-induced alterations at the organ level. This is consistent with the findings of El Ayed et al. [23].

### 3.2. Biochemical Indicator Analysis Results

**Figure 2:**
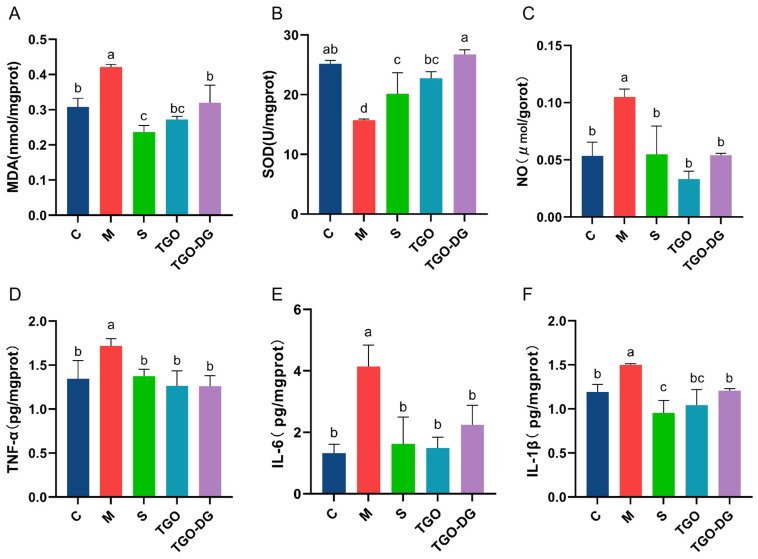
Effects of high-fat diet on lung lipid accumulation-related indicators in mice: oxidative stress markers (**A**) MDA; (**B**) SOD; (**C**) NO; inflammatory markers (**D**) TNF-α; (**E**) IL-6; (**F**) IL-1β. Different letters within the same column indicate statistically significant differences (*p* < 0.05).

TGO and TGO-DG modulated oxidative stress levels in the lung tissue of HFD-fed mice to different extents. As shown in Figure 2A–C, MDA and NO levels were significantly elevated (*p* < 0.05) in the M group compared with the C group, while SOD activity was markedly decreased (*p* < 0.05). These results suggest that HFD feeding induced pronounced oxidative stress in lung tissue, consistent with previous reports linking obesity to impaired pulmonary redox homeostasis. Ma et al. demonstrated that lipopolysaccharide exposure in HFD-fed mice induces significant pulmonary dysfunction, tissue injury, inflammation, and oxidative stress [24]. Compared with the M group, the MDA and NO levels in the TGO and TGO-DG groups were significantly reduced (*p* < 0.05), suggesting attenuation of lipid peroxidation and excessive NO production. In addition, the SOD level was significantly increased (*p* < 0.05) in the TGO-DG group compared with the M group, while the TGO group also exhibited an elevated SOD level, although to a lesser extent. These results suggest that TGO and TGO-DG reduce HFD-induced lung inflammation. The stronger effect of TGO-DG on antioxidant enzyme activity may be related to its distinct metabolic characteristics as a structured lipid, which is preferentially oxidized rather than stored. Wang et al. also found that diester oil intake is safe and, as a functional ingredient, can reduce body weight, stimulate visceral lipid metabolism, and counteract obesity [25].

Pulmonary inflammation was further evaluated to determine whether the observed redox modulation was accompanied by changes in inflammatory status [26]. As shown in Figure 2D–F, revealed pulmonary inflammation levels were significantly higher in the M group than in the C group (*p* < 0.05), indicating marked lung inflammation induced by HFD. These results support the notion that oxidative stress and inflammation are closely interconnected in obesity-associated lung injury [27]. In contrast, both TGO and TGO-DG treatment significantly reduced pulmonary inflammation marker levels compared with the M group (*p* < 0.05). No significant differences were observed between the TGO group or the TGO-DG group and the S group treated with lovastatin (*p* < 0.05), indicating that their anti-inflammatory effects were comparable. However, the TNF-α and IL-1β levels in the TGO-DG group were closer to those in Group C.

The results suggested that dietary intervention with TGO and TGO-DG alleviated HFD-induced oxidative stress and inflammatory responses in mouse lung tissue. The differential effects observed between TGO and its diester derivative highlighted the importance of lipid structure and metabolic fate in determining biological activity [28]. Structurally, triglycerides (TAG) consist of three fatty acids and are the primary form of fat stored in the body; in contrast, diglycerides (DAG) consist of only two fatty acids. This structural difference leads to distinct metabolic pathways. Therefore, in terms of energy utilization, once effective 1,3-diglycerides are digested, they cannot be resynthesized into fat for storage in the body like triglycerides; instead, they are directly used by the body for energy through oxidation and are thus considered less likely to cause fat accumulation. Research has also found that replacing triacylglycerols with diacylglycerols in high-fat diets can protect liver homeostasis by maintaining endoplasmic reticulum function, which is preserved through the inhibition of increased lysophosphatidylcholine production [8]. These findings suggest that TGO-DG is a promising dietary component worthy of further exploration for supporting respiratory health in the context of obesity-related metabolic dysfunction.

### 3.3. Effects of TGO and TGO-DG on the Expression of Inflammation-Related Genes and Proteins in Mouse Lungs

To elucidate the molecular mechanisms underlying the protective effects of TGO and TGO-DG against HFD-induced pulmonary inflammation, the PPAR-γ/NF-κB p65/IL-6 signaling pathway was investigated. PPAR-γ is a well-recognized anti-inflammatory nuclear receptor that negatively regulates NF-κB activation, thereby suppressing the transcription of pro-inflammatory cytokines such as interleukin-6 (IL-6) [29,30]. Various infections trigger a pro-inflammatory cytokine storm, with IL-6 as a major contributor, leading to diffuse alveolar damage in patients. However, the metabolic regulatory mechanisms of IL-6 in lung injury remain unclear [31,32]. Accordingly, mRNA expression levels of PPAR-γ, NF-κB p65, and IL-6 in lung tissue were determined by RT-PCR.

**Figure 3:**
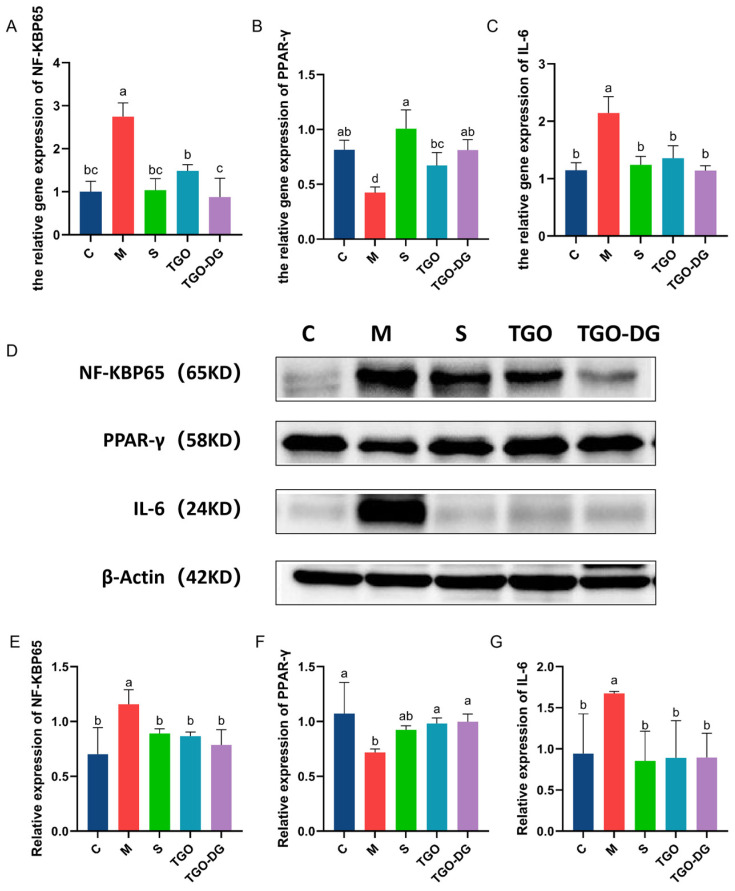
Expression of Inflammation-Related Genes in the Lungs of High-Fat Diet Mice (**A**) PPAR-γ; (**B**) NF-κB p65; (**C**) IL-6; (**D**) Effects of high-fat diet on NF-κB p65/PPAR-γ/IL-6 protein expression in lung inflammation, (**E**) NF-κB p65, (**F**) PPAR-γ, (**G**) IL-6. Data within the same column sharing the same letter indicate significant differences (*p* < 0.05).

As shown in Figure 3A–C, PPAR-γ mRNA expression was significantly reduced in the M group compared with the C group (*p* < 0.05), whereas NF-κB p65 and IL-6 mRNA levels were markedly elevated (*p* < 0.05), indicating activation of pro-inflammatory signaling in the lungs following HFD feeding. Compared with the M group, both TGO and TGO-DG treatment significantly upregulated PPAR-γ mRNA expression (*p* < 0.05) and concomitantly downregulated NF-κB p65 and IL-6 mRNA expression (*p* < 0.05). Notably, the TGO-DG group exhibited a more pronounced regulatory effect on these gene expression profiles than the TGO group, suggesting enhanced modulation of inflammatory signaling at the transcriptional level.

Consistent with the mRNA results, protein expression analysis further supported involvement of the PPAR-γ/NF-κB p65/IL-6 pathway in HFD-induced lung inflammation. As shown in Figure 3D–G, PPAR-γ protein expression was markedly decreased in the M group compared with the C group, whereas NF-κB p65 and IL-6 protein levels were significantly increased. Intervention with TGO or TGO-DG was associated with increased PPAR-γ protein expression and reduced NF-κB p65 and IL-6 levels. Among the treatment groups, TGO-DG showed the most significant upregulation of PPAR-γ protein expression and the strongest suppression of NF-κB p65 activation relative to the M group.

These results suggest that HFD-induced pulmonary inflammation is characterized by suppression of the anti-inflammatory regulator PPAR-γ and activation of the NF-κB/IL-6 pro-inflammatory axis at both the gene and protein levels in mice [33,34]. Dietary intervention with TGO and TGO-DG was associated with modulation of this imbalance, which may contribute to attenuation of pulmonary inflammatory responses. The superior efficacy observed in the TGO-DG group further suggests that lipid structure and metabolic characteristics may influence the regulatory capacity of these oils on inflammatory signaling pathways. Unlike TGO, which consists predominantly of triacylglycerols (TAG), TGO-DG is enriched in diacylglycerols (DAG). DAGs are preferentially oxidized rather than stored in adipose tissue, potentially reducing ectopic lipid accumulation in the lung and enhancing the bioavailability of bioactive fatty acids such as sciadonic acid (see Supplementary Table S2). Sciadonic acid, a Δ5-olefinic acid unique to Torreya seeds, has been shown to exert anti-inflammatory effects by modifying membrane phospholipid composition and suppressing NF-κB signaling. Thus, the combination of DAG structure and sciadonic acid enrichment may explain the superior efficacy of TGO-DG. Overall, these results suggest that *Torreya grandis*-derived oils play a protective role in alleviating obesity-associated lung injury, likely through regulation of the PPAR-γ/NF-κB p65/IL-6 pathway, and offer a promising nutritional strategy against obesity-associated lung injury.

### 3.4. Composition and Diversity Analysis of Lung Microbiota in High-Fat-Diet Mice

**Figure 4:**
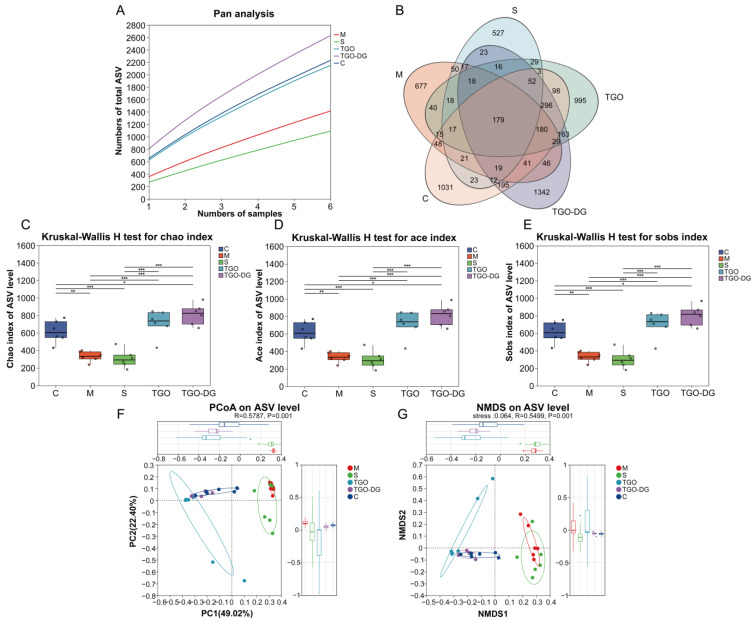
Analysis of lung microbiota ASV abundance and species composition (**A**) Pan_core species curve, (**B**) Species Venn diagram alpha diversity, (**C**) Chao index, (**D**) Ace index, (**E**) Sobs index beta diversity, (**F**) PCoA analysis plot, (**G**) NMDS analysis plot. Columns labeled with different letters indicate significant differences, (*p* < 0.05), * *p* < 0.05, ** *p* < 0.01, *** *p* < 0.001.

Amplicon sequence variants (ASVs) were used to assess microbial richness and diversity in mouse lung samples, with a higher ASV count reflecting greater microbial diversity [35]. As shown in Figure 4A, the cumulative ASV count of all groups gradually increased and reached a plateau with the increasing number of samples, suggesting that the sequencing depth was sufficient to capture the majority of microbial diversity present in the lung microbiota. Notably, the total ASV counts in the TGO, TGO-DG, and C groups were significantly higher than those in the M group, suggesting that HFD feeding was associated with a reduction in lung microbial diversity, while intervention with TGO or TGO-DG partially restored microbial richness.

The Venn diagram in Figure 4B further illustrates the distribution of shared and unique ASVs among different groups. The total numbers of ASVs detected in the C, M, S, TGO, and TGO-DG groups were 1031, 677, 527, 995, and 1342, respectively, indicating significant differences in lung microbial community composition across groups. Among them, the TGO-DG group had the highest number of ASVs, suggesting the richest lung microbial community in this group. A total of 179 ASVs were shared among all five groups, representing the core lung microbiota. Pairwise comparisons revealed that 48 ASVs were shared between the C and M groups, 23 between the C and S groups, and 195 between the C and TGO-DG groups. In addition, the TGO group and TGO-DG group shared 163 ASVs, whereas the TGO and M groups shared 40 ASVs, and the TGO-DG and M groups shared 46 ASVs.

These results suggest that HFD feeding markedly altered the composition and richness of the lung microbiota, whereas supplementation with TGO or TGO-DG shifted the microbial community structure toward that of the normal control group. The substantial overlap in ASVs between the TGO and TGO-DG groups suggests that both oils exert similar modulatory effects on lung microbiota composition, although TGO-DG appeared to promote a higher level of microbial richness. Given the emerging role of the lung microbiota in pulmonary immune homeostasis, the observed restoration of microbial diversity may be associated with the alleviation of HFD-induced pulmonary inflammation [36,37]. However, further studies are warranted to clarify the functional implications of these microbial changes and their causal relationship with lung injury.

Alpha diversity analysis was performed using the Chao index, Ace index, and Sobs index [38]. These indices reflect community richness, with higher values indicating greater microbial diversity. As shown in Figure 4C–E, the Chao index, Ace index, and Sobs index were higher in the C, TGO, and TGO-DG groups than in the M group. Notably, the TGO-DG group exhibited a significantly higher value than the M group, suggesting that *Torreya grandis* diester oil enhances microbial diversity in the lungs of high-fat-diet mice.

Principal coordinate analysis (PCoA) was employed to examine differences in beta diversity of mouse lung microbiota. As shown in Figure 4F,G, PCoA and NMDS plots revealed relatively large distances between samples from groups C and M, with these two groups exhibiting overall dispersion while other groups remained relatively clustered. This suggested a distinct separation in lung microbiota distribution between groups C and M. At the PC1 level, the distance between the TGO and TGO-DG groups and the C group decreased, suggesting significant changes in the composition of the mouse lung microbiota. Intervention with *Torreya grandis* oil and *Torreya grandis* diester oil was associated with restoration of the imbalance in the lung microbiota induced by a high-fat diet.

### 3.5. Analysis of Lung Microbiota at the Genus and Species Levels in High-Fat-Diet Mice

**Figure 5:**
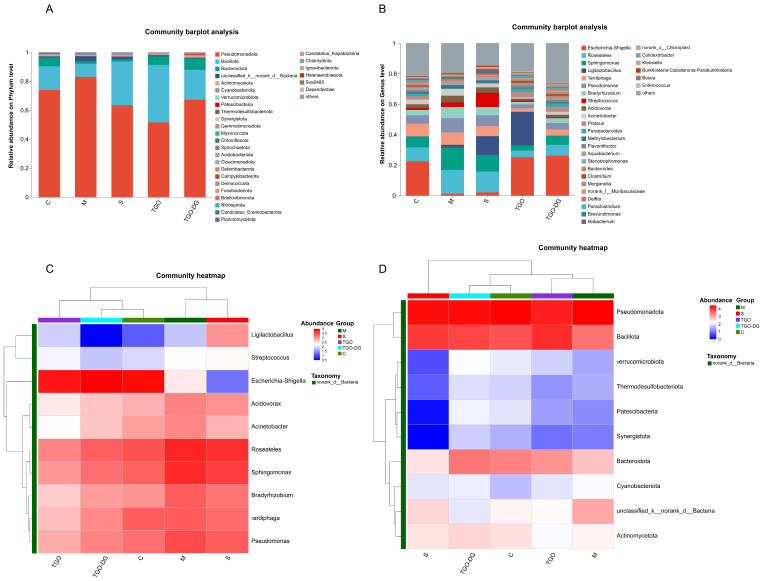
Community Composition Analysis. (**A**) Community Bar Chart at Family Level, (**B**) Community Bar Chart at Genus Level, (**C**) Community Heatmap at Family Level, (**D**) Community Heatmap at Genus Level.

To identify bacterial groups exhibiting differences between groups, species-level differential analysis was performed. Figure 5A presents the community bar plot analysis at the phylum level. *Pseudomonadota* was the most abundant phylum in all groups, followed by *Ascomycota* and *Bacteroidota*. *Bacillota* and *Bacteroidota* also showed significant representation across samples. The relative abundance of *Bacteroidota* was higher in the C, TGO, and TGO-DG groups than in the M group, suggesting that HFD feeding reduced pulmonary *Bacteroidota*, while TGO and TGO-DG interventions partially restored its abundance.

At the phylum level, dominant phyla such as *Pseudomonadota*, *Bacillota*, and *Bacteroidota* each formed distinct clusters, reflecting their specific abundance patterns. The community composition patterns of C, TGO, and TGO-DG samples were similar to each other, while those of the M and S groups were significantly separated, suggesting the uniqueness of community structure at the phylum level.

At the genus level, *Escherichia-Shigella*, *Roseateles*, *Sphingomonas*, *Coldextractor*, and others showed significant proportions across samples, revealing more refined differences than those at the phylum level. As shown in the heatmap clustering tree in Figure 5D, genera affiliated with *Pseudomonadota* and *Bacillota* (e.g., *Escherichia-Shigella*, *Sphingomonas*) formed distinct clusters, further reflecting differences in abundance patterns at the genus level.

*Ligilactobacillus*, a key beneficial genus that plays important roles in maintaining respiratory microbial balance, showed a significant increase in abundance following lovastatin intervention. Its relative abundance was highest in the TGO group, suggesting an association between TGO and enrichment of this beneficial genus. The increased presence of *Ligilactobacillus* correlated with restoration of pulmonary homeostasis, potentially via suppression of harmful bacteria and reduction of oxidative stress and inflammation (though these relationships remain correlational). These results suggest that intervention with TGO and TGO-DG modulated the lung microbial ecosystem, ameliorated HFD-induced microecological dysbiosis, and promoted the recovery of pulmonary microbial structure and function.

**Figure 6:**
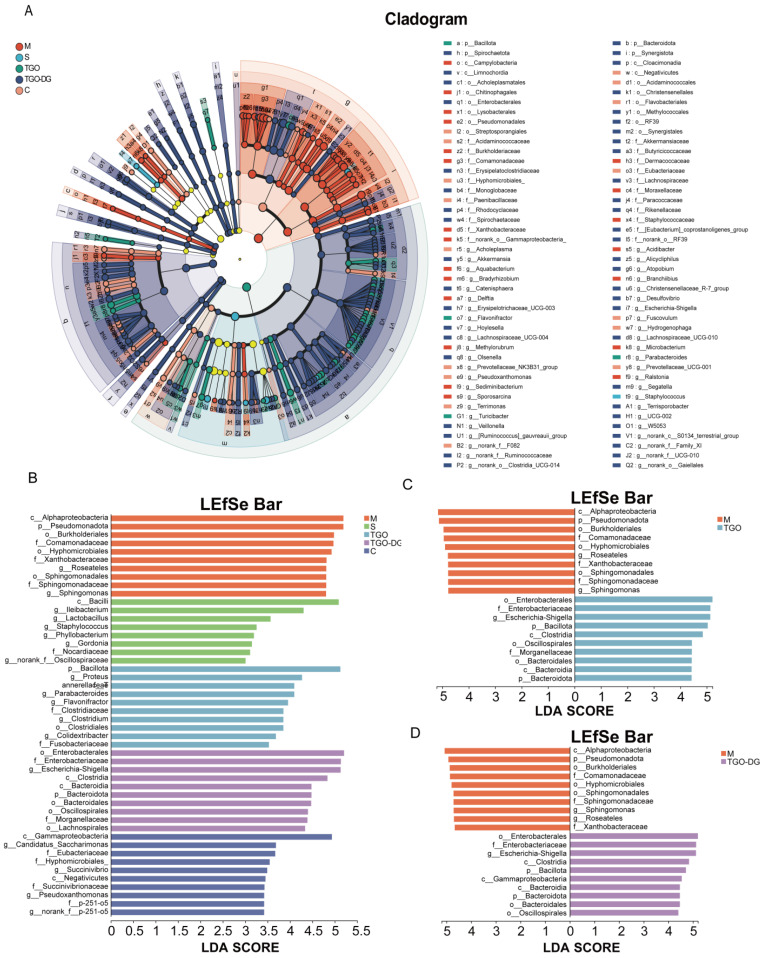
Species Differentiation Analysis. (**A**) LEfSe multi-level species hierarchy tree, (**B**) LDA discriminant bar chart, (**C**) LDA discriminant bar chart of lung microbiota between M and TGO groups, (**D**) LDA discriminant bar chart of lung microbiota between M and TGO-DG groups. Each column labeled with different letters indicates significant differences (*p* < 0.05).

To further identify taxa responsible for the differences in lung microbial communities among groups, LEfSe analysis was performed with an LDA score threshold of >3.5. As shown in Figure 6A,B, 5 specific microbial clusters exhibiting significant differential abundance from phylum to genus levels were identified across the experimental groups. In the control (C) group, taxa including *Gammaproteobacteria* and *Candidatus Saccharimonas* (*g_Candidatus_Saccharimonas*) were significantly enriched, serving as characteristic microbial markers for the C group. The M group exhibited pronounced enrichment of taxa associated with *Alphaproteobacteria* (*c_Alphaproteobacteria*), *Pseudomonadota phylum* (*p_Pseudomonadota*), *Burkholderiales* order (*o_Burkholderiales*), and *Comamonadaceae* family (*f_Comamonadaceae*), all of which displayed high LDA scores. These taxa collectively constituted the dominant microbial biomarkers of the M group, indicating that HFD markedly reshaped the lung microbiota toward a Proteobacteria-dominated profile, which is often associated with inflammation and dysbiosis. In the S group, differential taxa were mainly enriched within *Bacilli* (*c_Bacilli*), *Lellabacter* (*g_Lellabacter*), and *Lactobacillus* (*g_Lactobacillus*). These taxa served as core biomarkers for the S group and may reflect alterations in microbial composition induced by the corresponding intervention. Notably, the TGO group showed significant enrichment of *Phylum Bacillota* (*p_Bacillota*, *formerly Firmicutes*), as well as the genera *Proteus* (*g_Proteus*) and *Parabacteroides* (*g_Parabacteroides*). Similarly, in the TGO-DG group, taxa including Phylum Enterobacteriales (*o_Enterobacterales*), genera *Escherichia-Shigella* (*g_Escherichia-Shigella*), and class *Bacteroidetes* (*c_Bacteroidetes*) ranked highly in LDA scores, indicating that these taxa served as key differential microbial markers for this group.

As shown in Figure 6C,D, the diagram visually illustrates the core differences in microbial community composition between the M group and the TGO group and the TGO-DG group. The dominant taxa in the M group were primarily concentrated in *α-Proteobacteria* class and *Pseudomonadales*, whereas those in the TGO and TGO-DG groups were mainly distributed among *Enterobacteriales* order, *Ascomycota* phylum, and *Bacteroidetes* phylum, indicating a pronounced shift in microbial dominance following intervention.

These differentially enriched taxa can serve as potential microbial markers to distinguish C, M, S, TGO, and TGO-DG groups, providing key targets for subsequent studies on their functions (e.g., metabolic pathways, pathogenic/probiotic mechanisms) or group associations (e.g., disease versus health, treatment versus control) [39]. These findings further support the role of TGO and TGO-DG in modulating lung microbiota dysbiosis induced by a high-fat diet [40].

## 4. Conclusions

This study provides evidence for the protective effects of TGO and TGO-DG against lung inflammation in the HFD-induced model. Both TGO and TGO-DG interventions effectively alleviated HFD-induced pulmonary oxidative stress and inflammatory responses. TGO-DG showed superior protective efficacy compared to TGO, particularly in enhancing antioxidant defense and modulating the PPAR-γ/NF-κB/IL-6 signaling pathway, suggesting that the structural modification of TGO to TGO-DG enhances its bioactivity. Mechanistically, these protective effects were associated with the following: (1) a significant enhancement of antioxidant defense capacity, as evidenced by increased SOD activity and reduced MDA and NO levels in lung tissue; (2) effective inhibition of pro-inflammatory factors, including TNF-α, IL-6, and IL-1β; (3) modulation of the PPAR-γ/NF-κB/IL-6 signaling pathway, characterized by upregulation of PPAR-γ expression and suppression of NF-κB activation at both the transcriptional and translational levels; (4) enrichment of beneficial bacterial taxa, with both TGO and TGO-DG significantly ameliorating high-fat diet-induced pulmonary microbiota dysbiosis by improving microbial richness and diversity.

We did not measure systemic metabolic parameters such as blood glucose, insulin, or lipid profiles. Therefore, whether the observed pulmonary benefits are mediated directly by local effects of TGO/TGO-DG on lung tissue or indirectly through systemic metabolic improvements remains to be determined. Additionally, we did not perform histopathological examination of lung tissue (e.g., H&E staining) to directly visualize inflammation and tissue damage, nor did we conduct untargeted metabolomic profiling to identify potential bioactive metabolites of TGO and TGO-DG. Moreover, the observed associations between oil intervention and lung microbiota changes do not establish causality; future studies using germ-free mice or fecal microbiota transplantation are needed to determine whether microbial shifts contribute to the protective effects.

In summary, TGO and TGO-DG possess considerable potential as functional food ingredients for the prevention or mitigation of HFD-associated lung inflammation. This work provides a scientific basis for the further development and utilization of Torreya grandis-derived lipids in functional food and nutritional intervention strategies.

## Data Availability

The original contributions presented in this study are included in the article and Supplementary Materials. Further inquiries can be directed to the corresponding authors.

## Supplementary Materials

The following supporting information can be downloaded at https://www.mdpi.com/article/10.3390/nu18111671/s1. Table S1: Primer Sequences; Table S2: Fatty acid content of Torreya oil DAG (%). File S1: Characterization of the composition of TGO and GO-DG.
