# Supplementary material for: Torreya grandis Diester Oil Attenuates High-Fat Diet-Induced Pulmonary Inflammation with Superior Efficacy to Natural Torreya grandis Oil
Source: Nutrients. 2026 May 23;18(11):1671. doi: 10.3390/nu18111671 (PMC13259340; doi:10.3390/nu18111671)
Supplement: Supplementary file 1 [file nutrients-18-01671-s001.zip › nutrients-4293616-supplementary.pdf]

Table S1: Primer Sequences

gene	Primer sequence	
	Forward primer	Reverse primer
PPAR γ	TTCAAGGGTGCCAGTTTCG	CCATCTTTATTCATCAGGGAGG
NF- κ Bp65	GCCTCTGGCGAATGGCTTTA	GAGGGGAAACAGATCGTCCA
IL-6	TGATGGATGCTACCAAACTGGA	TGTGACTCCAGCTTATCTCTTGG
β-actin	AGTGTGACGTTGACATCCGT	GCAGCTCAGTAACAGTCCGC

Table S2. Fatty acid content of Torreya oil DAG (%)

Fatty acid	Crude oil	TAG	FFA	DAG	MAG
Palmitic acid	7.54	12.57	43.35	10.16	21.24
Stearic acid	3.41	8.62	41.79	6.93	17.65
Oleic acid	38.82	36.94	6.29	38.64	28.24
Linoleic acid	38.03	32.71	4.87	34.66	25.87
Linolenic acid	1.04	0.45	0.05	0.49	0.51
20:2	0.79	0.65	0.43	0.31	0.33
20:3 (8,11,14)	1.81	1.57	0.42	1.70	1.37
Sciadonic acid	8.27	6.49	2.81	7.10	4.80

TAG: triacylglycerol; FFA: free fatty acid; DAG: diacylglycerol; MAG: monoacylglycerol.

Characterization of the composition of TGO and TGO-DG

Experimental Report 001

Experiment Title: Enzymatic Catalysis of Glycerol Hydrolysis for the Preparation of Torreya Oil Diglycerides

Experiment No.: 001

Operator/Date: November 9, 2023

Abstract:

Gas chromatography was used to analyze the yield of diacylglycerol (DAG) from Torreya oil prepared by lipase-catalyzed glycerol hydrolysis.

I. Introduction and Objectives

To study and analyze the yield of DAG from Torreya oil prepared by lipase-catalyzed glycerol hydrolysis, verify the feasibility of preparing DAG from Torreya oil via enzymatic glycerol hydrolysis, and provide a basis for subsequent experimental analysis.

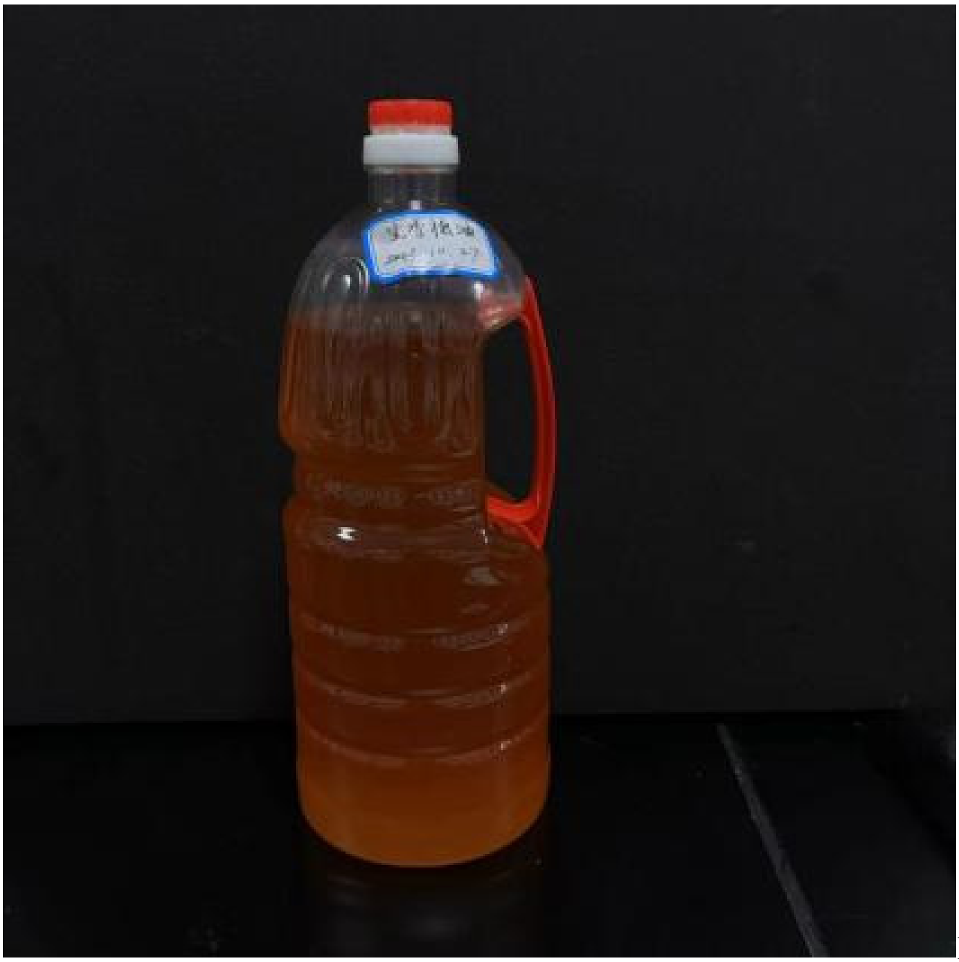

II. Materials and Methods

Materials and Reagents

Name	Specification	Manufacturer
Acetone	Chromatographic pure	Guangzhou Jinhua Large Chemical Reagent Co., Ltd.
n-Hexane	Analytical pure	Guangzhou Chemical Reagent Factory
Torreya oil	\	\
Glycerol	\	Tianjin Damao Chemical Reagent Factory

Name	Model	Manufacturer

Instruments and Equipment

Name	Model	Manufacturer
Gas chromatograph	Agilent 7820 A	Agilent Technologies, USA
Electronic balance	HR-120	Mettler Toledo
Constant temperature water bath	HR-4	Jintan Honghua Instrument Factory
Magnetic stirrer	IKA C-MAG HS 7	Zhengzhou Yingyu Yuhua Instrument Co., Ltd.
Rotary evaporator	HC-2518	Zhongjia Branch,科大创新股份有限公司
High-speed centrifuge	\	\

III. Results and Discussion

Table 1. Component contents of crude Torreya oil and the DAG product from glycerol hydrolysis (%)

Sample	FFA (%)	MAG (%)	DAG (%)	TAG (%)
Crude oil	0.54	0.47	0.78	98.20
Torreya oil DAG	0.38	0.69	90.94	7.99

As shown in Table 1, after molecular distillation of the lipase-catalyzed glycerol hydrolysis product, most of the FFA and MAG were removed, yielding a Torreya DAG oil containing 0.38% FFA, 0.69% MAG, 90.94% DAG, and 7.99% TAG.

Experimental Report 002

Experiment Title: Fatty Acid Composition Analysis of Torreya Oil Diglycerides
Experiment No.: 002
Operator/Date: November 15, 2023

Abstract:
Thin-layer chromatography was used to separate and analyze the fatty acid composition of each component in the Torreya oil diglycerides prepared by lipase-catalyzed glycerol hydrolysis in Experiment 001.

I. Introduction and Objectives

To study and analyze the fatty acid composition of each component in Torreya diglyceride oil prepared by lipase-catalyzed glycerol hydrolysis, with a focus on the proportion of sciadonic acid in each component.

II. Materials and Methods

Materials and Reagents

Name	Specification	Manufacturer
Ethyl ether	Chromatographic pure	Guangzhou Jinhua Large Chemical Reagent Co., Ltd.
n-Hexane	Analytical pure	Guangzhou Chemical Reagent Factory
Torreya diglyceride oil	\	\

Instruments and Equipment

Name	Model	Manufacturer
Gas chromatograph	Agilent 7820 A	Agilent Technologies, USA
Electronic balance	HR-120	Mettler Toledo
Constant temperature water bath	HR-4	Jintan Honghua Instrument Factory
Magnetic stirrer	IKA C-MAG HS 7	Zhengzhou Yingyu Yuhua Instrument Co., Ltd.
Rotary evaporator	RV 10 auto pro V-C	IKA, USA
High-speed centrifuge	HC-2518	Zhongjia Branch,科大创新股份有限公司

III. Results and Discussion

Table 2. Fatty acid content of Torreya oil DAG (%)

Fatty acid	Crude oil	TAG	FFA	DAG	MAG
Palmitic acid	7.54	12.57	43.35	10.16	21.24
Stearic acid	3.41	8.62	41.79	6.93	17.65
Oleic acid	38.82	36.94	6.29	38.64	28.24

Fatty acid	Crude oil	TAG	FFA	DAG	MAG
Linoleic acid	38.03	32.71	4.87	34.66	25.87
Linolenic acid	1.04	0.45	0.05	0.49	0.51
20:2	0.79	0.65	0.43	0.31	0.33
20:3 (8,11,14)	1.81	1.57	0.42	1.70	1.37
Sciadonic acid	8.27	6.49	2.81	7.10	4.80

TAG: triacylglycerol; FFA: free fatty acid; DAG: diacylglycerol; MAG: monoacylglycerol.

As shown in Table 2, in the Torreya oil prepared by the enzymatic method, sciadonic acid is mainly present in DAG and TAG, at 7.10% and 6.49% respectively, slightly lower than in the crude oil. In the FFA fraction after synthesis, palmitic acid and stearic acid are the major fatty acids. Sciadonic acid content is lower in MAG and FFA, at 4.80% and 2.81%, respectively.
